# Loss of Bone Mineral Density Associated with Age in Male Rats Fed on Sunflower Oil Is Avoided by Virgin Olive Oil Intake or Coenzyme Q Supplementation

**DOI:** 10.3390/ijms18071397

**Published:** 2017-06-29

**Authors:** Alfonso Varela-López, Julio J. Ochoa, José M. Llamas-Elvira, Magdalena López-Frías, Elena Planells, Lorenza Speranza, Maurizio Battino, José L. Quiles

**Affiliations:** 1 Institute of Nutrition and Food Technology “José Mataix Verdú”, Biomedical Research Center, University of Granada, Avda del Conocimiento s.n., Armilla, 18016 Granada, Spain; alvarela@ugr.es (A.V.-L.); jjoh@ugr.es (J.J.O.); maglopez@ugr.es (M.L.-F.); elenamp@ugr.es (E.P.); 2 Department of Physiology, Faculty of Pharmacy, University of Granada, Calle del Prof. Clavera s.n., 18071 Granada, Spain; 3 Nuclear Medicine Service, Hospital Virgen de las Nieves, Avda. de las Fuerzas Armadas 2, 18014 Granada, Spain; josem.llamas.sspa@juntadeandalucia.es; 4 Department of Medicine and Science of Aging, University of Chieti “G. D’Annunzio”, 66100 Chieti, Italy; lorenza.speranza@unich.it; 5 Department of Scienze Cliniche Specialistiche ed Odontostomatologiche, Università Politecnica delle Marche, 60131 Ancona, Italy; m.a.battino@univpm.it

**Keywords:** bone mineral density, F_2_-isoprostanes, oxidative stress, MUFA, PUFA

## Abstract

The role of dietary fat unsaturation and the supplementation of coenzyme Q have been evaluated in relation to bone health. Male Wistar rats were maintained for 6 or 24 months on two diets varying in the fat source, namely virgin olive oil, rich in monounsaturated fatty acids, or sunflower oil, rich in n-6 polyunsaturated fatty acids. Both dietary fats were supplemented or not with coenzyme Q_10_ (CoQ_10_). Bone mineral density (BMD) was evaluated in the femur. Serum levels of osteocalcin, osteopontin, receptor activator of nuclear factor κB ligand (RANKL), osteoprotegerin (OPG), adrenocorticotropin (ACTH) and parathyroid hormone (PTH), as well as urinary F_2_-isoprostanes were measured. Aged animals fed on virgin olive oil showed higher BMD than those fed on sunflower oil. In addition, CoQ_10_ prevented the age-related decline in BMD in animals fed on sunflower oil. Urinary F_2_-isoprostanes analysis showed that sunflower oil led to the highest oxidative status in old animals, which was avoided by supplementation with CoQ_10_. In conclusion, lifelong feeding on virgin olive oil or the supplementation of sunflower oil on CoQ_10_ prevented, at least in part mediated by a low oxidative stress status, the age-related decrease in BMD found in sunflower oil fed animals.

## 1. Introduction

In humans, regardless of gender, aging leads to a progressive decline in bone mass, which is associated with other factors affecting bone architecture and organization [1,2,3]. These changes would explain the increased prevalence of osteoporosis in the aged population [4]. Osteoporosis has been defined as “a skeletal disorder characterized by compromised bone strength and predisposing to an increased risk of fracture” [5]. Bone fractures are responsible for a significant mortality and morbidity particularly notably in elderly persons [6]. The tremendous economic and healthcare burdens caused by osteoporotic fractures [7] makes it imperative to promote effective prevention and treatment strategies to counterbalance them [8]. Prevention must be focused not only in the female population, but also in men, where this pathology is also very important [9,10]. Nutritional factors play a role in skeletal health during aging. In that sense, the importance of calcium, vitamin D or protein intake is clearly due to the role of these nutrients in supporting bone matrix production and mineralization. In addition, recent evidence suggests that other dietary factors may influence bone homeostasis and may be important for long-term bone health [8]. However, other dietary components might be considered from the point of view of osteoporosis, like those endorsed with antioxidant and/or anti-inflammatory properties [11,12]. According to that, dietary fat might have a relevant role in bone aging. In fact, observational studies have offered some interesting associations between dietary fat intake and osteoporosis. Overall, high-fat diets, particularly those rich in saturated fats, have been associated with a lower bone mineral density (BMD) and a higher osteoporosis degree [13,14]. On the other hand, there is evidence for a positive effect of polyunsaturated fatty acids (PUFA) [15,16,17,18] and monounsaturated fatty acids (MUFA) on bone health [17,18,19]. However, results concerning the effect of these unsaturated fatty acids have been in some term contradictory [20,21,22,23].

Most of the research on dietary fat and bone health performed to date do not cover the full picture. First, research on the combined role of aging and dietary fat on bone health in males is scarce. Second, most of interventions have been performed on postmenopausal (i.e., ovarectomized) rodent models or directly on postmenopausal women. However, other causes of bone loss can be overimposed to the effects of sex steroid deficiency contributing to fracture risk. In that sense, an age-dependent decline of bone mass and strength in sex steroid-sufficient female or male mice has been also reported [24]. Third, regarding animal studies, most of them have been performed on very young individuals that have not reached skeletal maturity and consequently they did not reflect a good model of senile osteoporosis [7]. Fourth, from a nutritional standpoint, many interventions based on the fat component of the diet consisted in the supplementation with some types of fatty acids for relative short periods [16,17,25]. Most of them evaluated the effects of fatty acids on disease progression or recovery, particularly those performed in postmenopausal women. However, there are few data about the effect of dietary fat as a life-long preventive agent on bone features. Experimental studies considering fat in the whole dietary context are also scarce.

MUFA and n-6 PUFA have previously reported opposite consequences in different tissues during aging [26,27]. According to that, in the present study, long-lived male rats were used as an experimental model of aging to evaluate the role of different dietary unsaturated fats on bone aging and health. To do this, animals were fed life-long on purified, semisynthetic, normocaloric and normolipidic diets based on two different fat sources (virgin olive or sunflower oils) rich in unsaturated fatty acids but with clearly different lipid profiles (mainly rich in MUFA or n-6 PUFA, respectively). Moreover, in the past, it has been showed that coenzyme Q_10_ (CoQ_10_) supplementation was able to avoid in several tissues the deleterious effects associated with highly pro-oxidant fats, like those rich in n-6 PUFA [28,29,30]. Consequently, the interaction between dietary fat and CoQ_10_ at the bone level was investigated in the present study by including in the experimental design groups of rats fed on the different dietary fats with our without CoQ_10_ supplementation.

## 2. Results

### 2.1. Animal Weight

At the end of the experiment, rats from the V group (rats fed on virgin olive oil) presented higher, but not significantly different body weight (534.5 ± 51.8 g) than those fed on sunflower oil from the S group (animals fed on sunflower oil (508.3 ± 19.5 g). In the same way, no differences concerning body weight were found between animals fed on virgin olive oil plus CoQ_10_, i.e., the VQ (Virgin olive oil + CoQ_10_) group (496 ± 20 g) and those fed on sunflower oil plus CoQ_10_, i.e., the SQ (Sunflower oil + CoQ_10_) group (499.0 ± 6.6 g). From the observation of body weight evolution and food spillage, no differences concerning food intake were inferred between groups.

### 2.2. Plasma Fatty Acid Profile and Total Coenzyme Q Content

The sum of plasma MUFA levels (in g/100 g) for animals from the V group at six months of age was 40.6 ± 1.8, significantly higher (*p <* 0.05) than levels found for animals from the S group (27.9 ± 3.3). Concerning n-6 PUFA for six month old animals, the V group led to 19.8 ± 0.7 g/100 g, significantly lower (*p <* 0.05) than those found in the S group (32.2 ± 2.7 g/100 g). Such differences were maintained for old animals and irrespective of the CoQ_10_ supplementation in the diet (data not showed). Concerning plasma CoQ_10_, animals supplemented on CoQ_10_ for six months showed significantly higher concentration (*p <* 0.05) than their non-supplemented counterparts (in terms of µM concentration, values were 39.24 ± 9.25 vs. 14.99 ± 0.78, for VQ vs. V; and 50.71 ± 5.22 vs. 17.03 ± 0.77, for SQ vs. S). Differences were maintained or even increased for old animals (Material not intended for publication: Quiles, J.L. University of Granada, Granada, Spain. Old animals supplemented on CoQ_10_ showed higher values than the non-supplemented old rats, 2017).

### 2.3. Bone Mineral Density, Bone Mineral Content (BMC) and Bone Areal Size

**Figure 1:**
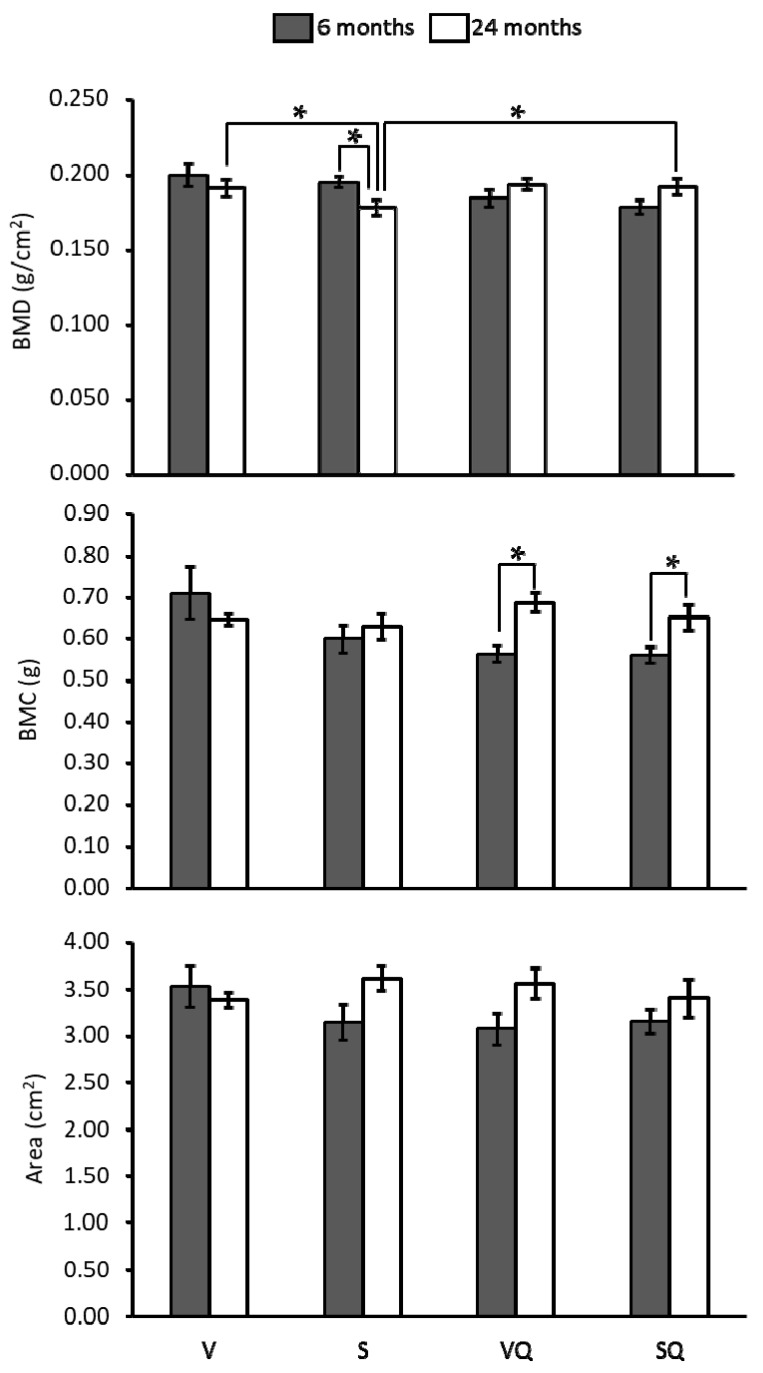
Effects of dietary fat and CoQ_10_ on bone mineral density (BMD), bone mineral content (BMC) and bone cross-sectional area in 6- and 24-month-old rats. Results are expressed as mean ± standard error of mean of six animals. Abbreviations: V, virgin olive oil; VQ, virgin olive oil + CoQ_10_; S, sunflower oil; SQ, sunflower oil + CoQ_10_. * Statistically significant differences (*p <* 0.05).

Results from BMD, BMC and bone cross-sectional area measurement of the proximal femur are presented in Figure 1. Regarding differences between non-supplemented dietary groups, the V group showed higher values than the S group, although only at 24 months. For supplemented groups, no differences between different groups were showed at any age. At 24 months of age, BMD was lower in animals fed on sunflower oil compared with those receiving similar diets supplemented with CoQ_10_. Concerning the aging effect, BMD was lower in old animals fed non-supplemented sunflower oil with respect to their younger counterparts. However, in animals receiving CoQ_10_, no significant age-related differences were observed. In turn, for BMC, only statistically significant differences were found between age groups for supplemented animals, with older animals showing the highest values. Lastly, no statistically significant differences were found for bone cross-sectional areas.

### 2.4. Urinary F_2_-Isoprostanes

**Figure 2:**
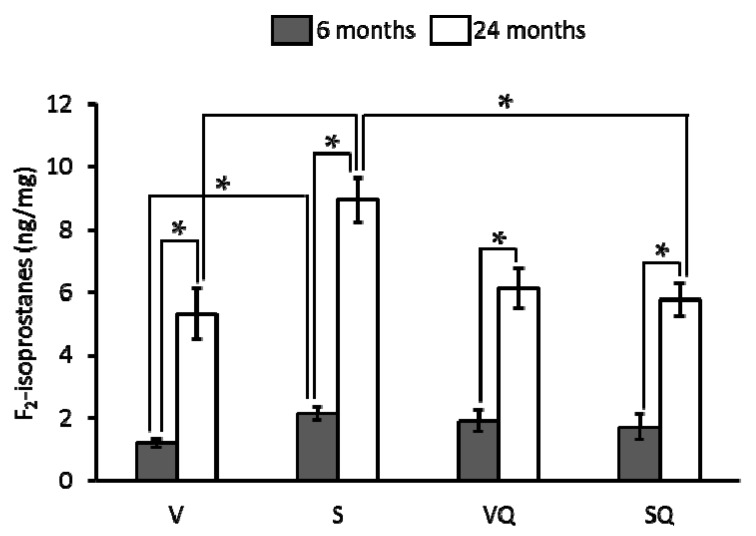
Effects of dietary fat and CoQ_10_ on F_2_-isoprostanes urinary levels in 6- and 24-month-old rats. Results are expressed as mean ± standard error of mean of six animals. Abbreviations: V, virgin olive oil; VQ, virgin olive oil + CoQ_10_; S, sunflower oil; SQ, sunflower oil + CoQ_10_. * Statistically significant differences (*p <* 0.05).

Urinary F_2_-isoprostanes levels are showed in Figure 2. Concerning diet effect, for 6-month-old animals fed on non-supplemented oils, the lowest values were found for virgin olive oil fed animals at both ages. In relation to animals fed on the CoQ_10_-supplemented diets, no differences were found between dietary groups at any age. All aged animals led to significantly higher levels than their younger counterpart.

### 2.5. Circulating Levels of Ostecalcin and Osteopontin

**Figure 3:**
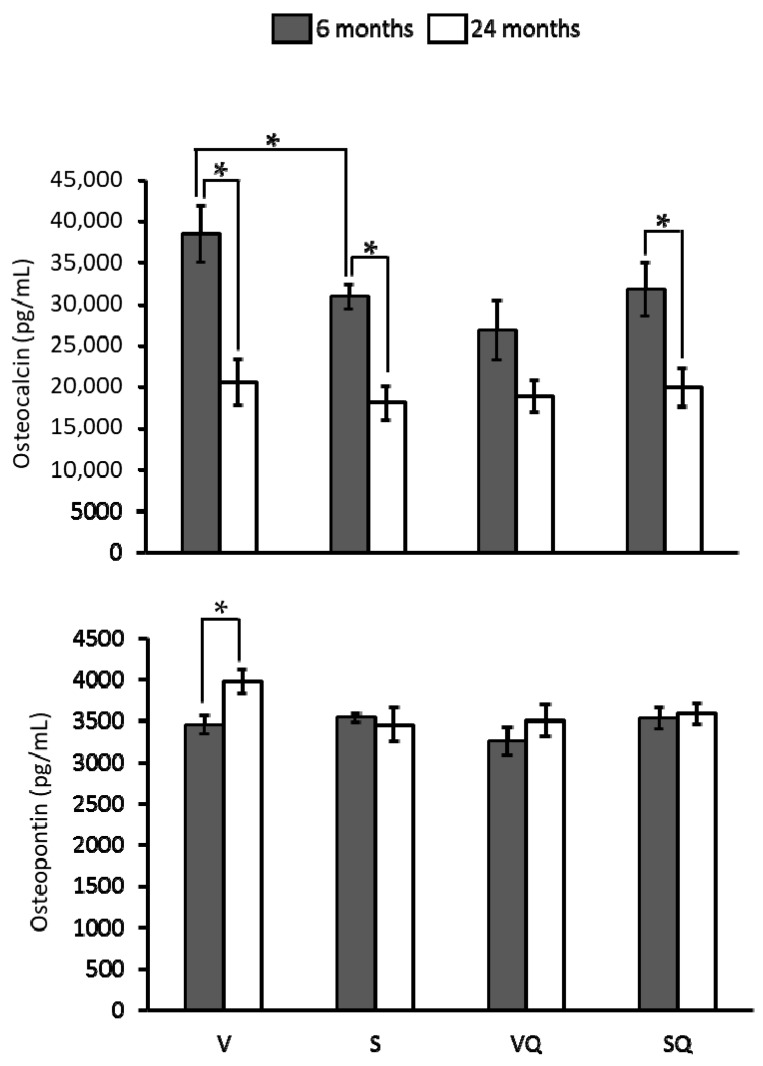
Effects of dietary fat and CoQ_10_ on circulating levels of osteocalcin and osteopontin in 6- and 24-month-old rats. Results are expressed as mean ± standard error of mean of six animals. Abbreviations: V, virgin olive oil; VQ, virgin olive oil + CoQ_10_; S, sunflower oil; SQ, sunflower oil + CoQ_10_. * Statistically significant differences (*p <* 0.05).

Osteocalcin levels (Figure 3) were lower in rats from the S group than in those fed from the V group at six but not at 24 months of age. There were no differences between groups maintained on CoQ_10_-rich diets at any age. Likewise, no significant differences were found between these groups and those fed on the same diets with CoQ_10_ at any age. In relation to age, it was noted that older animals presented the lowest values in all dietary groups except VQ. Regarding osteopontin (Figure 3), no significant differences were found among dietary groups at any age. Concerning differences between age groups, lower levels for this marker were found in young animals fed on virgin olive oil with respect to their older counterparts.

**Figure 4:**
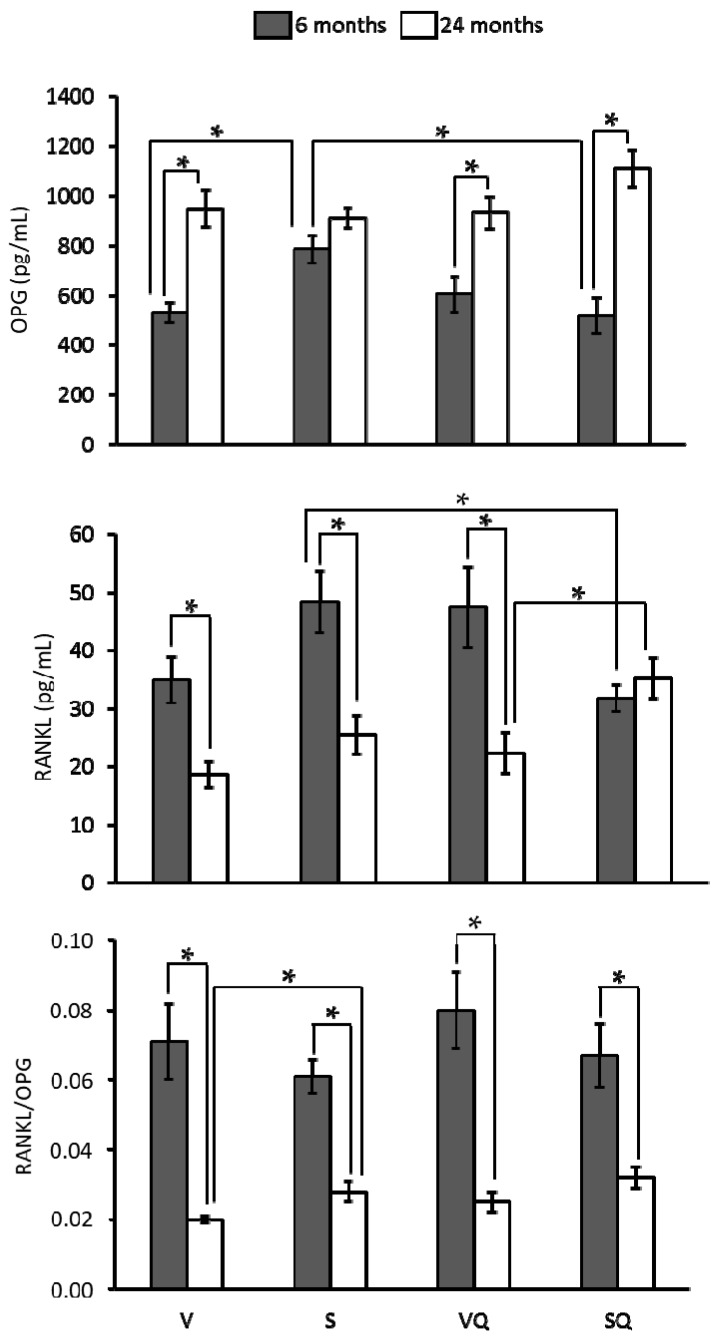
Effects of dietary fat and supplementation with coenzyme Q_10_ (CoQ_10_) on serum levels of osteoprotegerin (OPG), receptor activator of the nuclear factor κB ligand (RANKL), and RANKL/OPG ratio in 6- and 24-month-old rats. Abbreviations: V, virgin olive oil; VQ, virgin olive oil + CoQ_10_; S, sunflower oil; SQ, sunflower oil + CoQ_10_. * Statistically significant differences (*p <* 0.05).

Results from osteoprotegerin (OPG), receptor activator of nuclear factor κB ligand (RANKL) levels and RANKL/OPG ratio are presented in Figure 4. OPG levels were higher in older animals, whereas RANKL levels were lower. Exceptions were found in the S group for OPG and SQ for RANKL, where no differences associated with age were found. Regarding differences between dietary groups, lower OPG levels were found in the V group compared to the S group at six months of age. Similar differences were found for RANKL between supplemented animals at 24 months. Comparisons between supplemented and non-supplemented animals revealed lower levels of both markers at six months of age. Concerning the RANKL/OPG ratio, differences in relation to dietary groups or CoQ_10_ treatments were found only among old animals receiving non-supplemented diets, with the V group presenting a lower value than the S group. Regarding age effect, lower values were found for older animals in all groups.

### 2.6. Circulating Levels of Parathyroid Hormone (PTH) and Adrenocorticotropin (ACTH)

**Figure 5:**
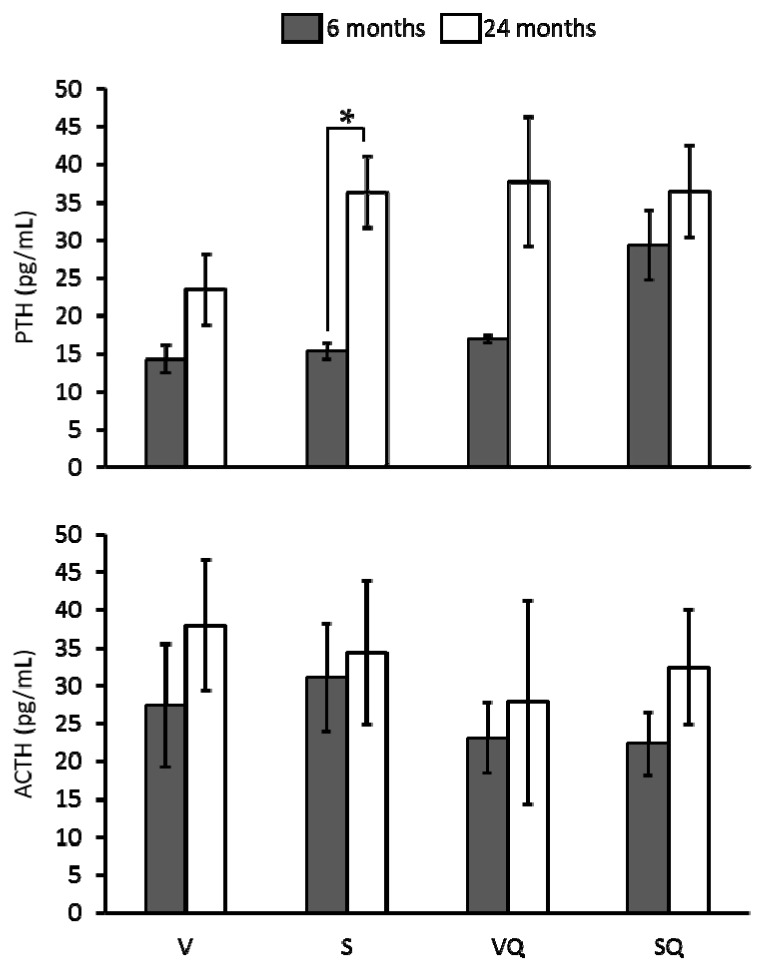
Effects of dietary fat and CoQ_10_ on parathyroid hormone (PTH) and adrenocorticotropin (ACTH) circulating levels in 6- and 24-month-old rats. Results are expressed as mean ± standard error of mean of six animals. Abbreviations: V, virgin olive oil; VQ, virgin olive oil + CoQ_10_; S, sunflower oil; SQ, sunflower oil + CoQ_10_. * Statistically significant differences (*p <* 0.05).

Figure 5 shows the results in relation to circulating level of analyzed hormones. Statistical analysis showed no significant differences between dietary groups in relation to ACTH and PTH serum levels at any age. Regarding age, a higher PTH level in old animals from the S group was found when compared with their younger counterparts.

## 3. Discussion

Aging is associated with bone loss and subsequent derived fractures [2,3]. From a preventive point of view, an adequate nutrition during life could result in reduction of such risk. Despite early interest being focused on calcium and vitamin D, there is no evidence that other dietary components such as lipids or antioxidants could also have implications on bone health. In the present study, rats were fed their entire lives on different dietary fats varying in their lipid profile, virgin olive oil (rich in MUFA), or sunflower oil (rich in n-6 PUFA). Both dietary fat types were administered with or without CoQ_10_ supplementation and BMD was evaluated at different ages. Firstly, verifying a proper adaptation to the diets is required to attribute any effect of these nutritional treatments on BMD. This was confirmed by the plasma fatty acid profiles and CoQ_10_ contents found in the animals of the present study. BMD is a major parameter determining bone strength, which is used for the diagnosis of osteoporosis and that is expected to decrease during aging [2,3]. Observational studies in the old segment of the population have indicated a positive association between PUFA intake and BMD [15,31]. Several studies evaluating n-3/n-6 PUFA ratio, or their respective levels have indicated a positive role for n-3 PUFA over n-6 PUFA [15,20,32]. Concerning studies on MUFA and BMD, information is scarce, but there is at least a study reporting a positive relationship [19]. Regarding animal studies, most have reported positive beneficial effects for n-3 PUFA [16,17], although there are some contradictory results [21]. In the present research, BMD analysis reported lower values for old animals fed on sunflower oil when compared with those fed on virgin olive oil despite no differences were observed in BMC. Thus, this would indicate that the use of virgin olive oil in a long-term dietary pattern would prevent BMD depletion associated with age in male subjects. These results agree with previous studies in ovariectomized rats reporting the potential of virgin olive oil for the prevention of osteoporosis [25].

For osteoporosis diagnosis in humans, individual BMD values are usually expressed in relation to a reference population in terms of standard deviation units to reduce calibration differences between instruments. Usually, these units are commonly used in relation to the young healthy population, referring experimental measures to the so-called T-score. In the present study, young animals belonging to the S group might be used as a reference to determine T-score. According to that, old animals receiving non-supplemented sunflower oil would be in the low bone mass category (osteopenia) according to the World Health Organization (WHO) proposal modified by the International Osteoporosis Foundation for men and women [33]. Following this approach, BMD values found for the V group at 24 and 6 months of age would belong to the normal category. Clinical significance of these categories lies in the fracture risk that arises and a strong gradient of risk has been reported for hip fracture and BMD determined by the dual energy X-ray absorptiometry (DXA), particularly at the proximal femur. Thus, it has a 2.6-fold increase in fracture risk has been described for each SD decrease in BMD [33]. Therefore, having a diet based on virgin olive oil as the main fat source, instead of sunflower oil, might be a good long-term strategy to prevent bone fractures and their derived problems. Virgin olive oil’s beneficial effect on bone health has been also suggested in humans, but only in a study performed in a group of subjects aged 30–50 years who had undergone hysterectomy. In that study, olive oil supplements prevented BMD losses in some lumbar vertebrae and femur [25], although control group did not receive any placebo. As it has been described in most of the previous interventions, known harmful factors for BMD (as inflammation or sex steroid deficiency) were experimentally induced or were present, which often are associated with aging, but they did not evaluate aging per se. In turn, our model reinforces some advantages of MUFA and supports the negative role of n-6 PUFA in BMD maintenance with aging, regardless of risk factor presence. In addition, the nutritional intervention performed in the present study involved the modification of all dietary fat content and not only a partial modification, a factor that also may help to explain some discrepancies found between supplementation studies.

Concerning the role of CoQ_10_, when the diets based on sunflower oil were supplemented with this molecule, the deleterious effect of aging on BMD was prevented. Interestingly, results suggest that dietary CoQ promoted an increase in BMC with age. Because BMD is the ratio of BMC to bone areal size, this contradiction between BMD and BMC values could be explained by changes in bone size, but neither significative change was found in this parameter. A preventive effect of CoQ on BMD loss has been also suggested by one study in rats where a short-term treatment with CoQ_10_ prevented spinal cord injury-induced loss of bone and mineral content [34]. Using the T-score criteria, as above, BMD found in SQ and VQ groups at 24 months may be considered normal. Therefore, long-life supplementation with CoQ_10_ has an interesting potential for the prevention of osteoporosis and the maintenance of bone strength under certain dietary habits, i.e., frequent consumption of n-6 PUFA-rich diets. Human Equivalent Dose (HED) for the dose administrated to these rats would be 0.26 mg/kg per day assuming a weight of 70 kg for an average man. This means that a 70 kg man would need 18.43 mg per day to reach such a value. In most of the interventions in humans, dietary CoQ is provided at very high doses (usually ranging from 100 to 2400 mg/day) for short periods of time for old or ill people [35]. In turn, the results of the present study would indicate that maintaining a lifelong CoQ low-dosage supplementation would have a preventive effect on the deleterious aspects of n-6 PUFA-rich diet consumption with respect to bone health during aging.

Because bone remodeling is also a major determinant of bone strength; serum levels of osteocalcin were measured to assess changes in bone turnover. Osteocalcin decreased with aging to similar levels for all diets, although virgin olive oil was associated with the highest values at six months. In an intervention trial, it has been observed that circulating osteocalcin was increased in men following a Mediterranean diet rich in virgin olive oil. Moreover, virgin olive oil consumption was positively associated with total osteocalcin at baseline and follow-up before and after adjusting for the use of statins. Osteocalcin is an extracellular matrix protein that is released into blood during both new matrix formation and existing matrix breakdown [36]. Thus, its serum levels can be indicative of bone turnover rate. According to this, it might be possible that MUFA promote a higher bone turnover rate in early life and/or the maintenance of this for a longer time, improving bone structure maintenance through life. Bone resorption rate has also been positively related to inflammation. Osteopontin plasma levels can offer an idea about the influence of a proinflammatory state or inflammatory disorders on bone health [37,38]. In the present study, higher osteopontin levels associated with age were found in rats from the V group. This could indicate that aged animals fed on virgin olive oil had a higher rate of bone resorption because of inflammatory processes. Alternatively, these results might mean that that young animals from this group had a lower resorption rate, since there were no significant differences with the other dietary groups at any age. Nevertheless, it would be necessary for a more specific marker to confirm the link between osteopontin and bone resorption. On the other hand, CoQ supplementation had no relevant effects on any of these markers.

As it is well known, several hormones can influence bone metabolism, and differences associated with age have been described for most of these hormones. According to that, PTH and ACTH were evaluated. For PTH, results showed higher values for aged rats fed on sunflower oil. This hormone has shown to exert a dual role in bone mass. On one hand, it is known that this hormone is released when calcium levels in blood are low to induce bone resorption process and to mobilize bone calcium into blood, so high levels of PTH were associated with higher bone resorption rate. On the other hand, it has been reported that moderate levels during short periods could even increase bone mass in spite of the punctual increases in resorption rate [39]. Here, it seems that the high levels of PTH in old animals fed on sunflower oil, probably maintained for a long period, can be related to a higher bone loss in these animals. Concerning ACTH, serum levels were similar for all studied groups, so no conclusion can be inferred from the study of this marker.

Progressive loss of bone strength and mass has been shown to be temporally associated with decreased osteoblast and osteoclast numbers and decreased bone formation rate in sex steroid-sufficient female or male mice, as well as with increased osteoblast and osteocyte apoptosis [40]. Here, the serum levels of two antagonist proteins involved in the key osteoclastogenesis regulatory step, RANKL and OPG, were also evaluated. RANKL is a membrane protein presented by osteoblasts and other bone cells, which binds to the RANK [41]. OPG is a soluble decoy receptor for RANKL that competitively antagonizes RANKL–RANK interaction, inhibiting osteoclastogenesis [41]. Results suggest that RANKL decreases with aging, whereas OPG increases in the S group regardless of CoQ presence in the diet. In turn, OPG levels would remain constant during aging in the S group despite the fact that RANKL would exhibit the same behavior as in the previous groups. It has been suggested that an age-associated increase in OPG could be important to protect bone from excessive resorption during aging [26,28]. Thus, it could be feasible that absence of variations in S group would lead to higher bone resorption in this group. However, it is important to take into account that these animals showed the highest levels of OPG at six months of age compared with the other dietary groups. Interestingly, dietary CoQ_10_ in addition to sunflower oil led to lower levels of RANKL in young rats, but this factor has a similar effect in the same sense over OPG levels. In addition, no effects were found in old animals. To go deeper into this issue, RANKL/OPG ratio was also evaluated. Results suggest that the serum RANKL/OPG ratio decreases with aging in all groups, as it has been previously reported [42]. Moreover, virgin olive oil led to higher levels in aged subjects than sunflower oil, whereas this difference was absent under CoQ_10_ supplementation. However, there is not any statistically significant effect of CoQ_10_ on RANKL/OPG ratio for dietary groups in this study.

The effect of both dietary fat and CoQ_10_ might be related to reactive oxygen species (ROS) roles on bone resorption. Several pieces of evidence link oxidative stress to BMD decrease in humans [43,44]. In addition, its association with age-dependent decline of bone mass and strength in sex steroid-sufficient female or male mice has been also reported [24]. Oxidative stress has shown to damage osteoblasts and to suppress their differentiation [45]. Moreover, ROS serves as signaling molecules in osteoclasts, enhancing their differentiation [46]. This is particularly interesting since NF-κB is an oxidative stress-responsive transcription factor and may be activated by free radicals, so intracellular levels of ROS would act as “secondary messengers” in osteoclast or osteoclast progenitor cells [46]. Therefore, the higher oxidative stress levels observed in aged rats, particularly when they have been fed on diets rich in polyunsaturated fat could led to an increase in sensibility to RANKL-stimulation explaining a higher osteoclastogenesis and bone resorption in spite of the age-associates decrease in RANKL levels. In RANKL-stimulated bone marrow-derived monocytes and RAW 264.7 cells, a model of an osteoclast, CoQ_10_ inhibited osteoclastogenesis [45,46]. Such an effect has been suggested to be a consequence of the ROS scavenging properties of this molecule [47]. In that sense, evidence suggests that CoQ_10_ strongly suppressed H_2_O_2_-induced IκBα, p38 signaling pathways [48]. Results from the present study concerning oxidative stress may support antioxidant properties of CoQ_10_, at least in part, and, consequently, the positive effects of this molecule on BMD. In fact, not only CoQ_10_, but also dietary fat affected oxidative stress levels. It has been widely described that dietary fat is able to modulate oxidative stress in rat models of aging, with MUFA leading to a lower oxidative level through aging than n-6 PUFA or n-3 PUFA based diets [26,27]. In addition, we have previously found that when sunflower oil is supplemented on CoQ_10_, oxidative stress was less prominent during aging [28,29,30,49]. Urinary F_2_-isoprostanes from the present study showed that aged animals fed on sunflower oil had the highest oxidative stress levels and that supplementation of this fat with CoQ_10_ prevented this fact, in parallel to the prevention of BMD decrease. Thus, oxidative stress might help to explain changes in BMD in sunflower oil fed animals, as well as the lowest loss in BMD found in virgin olive oil fed animals.

Summarizing, virgin olive oil prevented the age-related decrease in BMD found in sunflower oil fed animals. The reduction of BMD associated with age in animals fed on sunflower oil was prevented by CoQ_10_ supplementation. Oxidative stress might be in the cause of the lower BMD associated with age in virgin olive oil fed animals and in those fed on sunflower oil supplemented on CoQ_10_. Notwithstanding this, other aging-affected processes with consequences in bone health need to be further investigated in this type of aging model. Moreover, research must be extended to female individuals despite a more complex experimental design being required.

## 4. Materials and Methods

### 4.1. Experimental Design

Forty-eight male Wistar rats (*Rattus norvegicus*) weighing 80–90 g were housed three per cage and maintained in a 12-h light/12-h darkness cycle, with free access to food and water. The rats were randomly assigned into four experimental groups and fed from weaning until 24 months of age on a semi-synthetic and isoenergetic diet according to the AIN93 criteria [50] composed of (in g/100 g of diet): 14 casein, 46.57 starch, 10 sucrose, 15.5 dextrose, 4 dietary fat, 5 cellulose, 0.25 choline, 0.18 l-cystine, 1.0 vitamin mixture, and 3.5 mineral mixture. The experimental diets differed in two aspects. On one hand, two different dietary fat sources were used, virgin olive oil and sunflower oil, which are rich in MUFA or n-6 PUFA, respectively. On the other hand, an additional supplemented version of each diet was prepared to reach 2.5 mg/kg per day of CoQ_10_. Thus, four groups were established, virgin olive oil fed animals (V group), sunflower oil fed animals (S group), virgin olive oil plus CoQ_10_ fed animals (VQ group) and sunflower oil plus CoQ_10_ fed animals (SQ group). Six rats per group were killed at 6 and 24 months from the start of the experiment. The rats were killed by cervical dislocation followed by decapitation, at the same time of the day, to avoid any circadian fluctuation. Blood was collected in ethylene diamine tetraacetic acid (EDTA)-coated tubes, and plasma was centrifuged at 1750 g for 10 min. Femur bones were isolated from rats, weighted and preserved for further analyses. Urine was collected and stored at −80 °C until analyzed. The animals were treated in accordance with the guidelines of the Spanish Society for Laboratory Animals and the experiment was approved by the Ethical Committee of the University of Granada (permit number 20-CEA-2004, 10 April 2004).

### 4.2. Determination of Bone Mineral Density

Bone mineral content (BMC) and bone areal size were measured in the proximal half of the isolated femur bones by dual energy X-ray absorptiometry (DXA) using a Hologic QDR-4500 Elite densitometer (Hologic, Inc., Bedford, MA, USA). Then, BMD was calculated as bone mineral content (BMC)/area.

### 4.3. Urinary F_2_-Isoprostanes Determination

Total F_2_-isoprostanes were measured in urine by a competitive enzyme immunoassay (R&D Systems, Minneapolis, MN, USA). Results were normalized to urinary creatinine.

### 4.4. Determination of Bone Metabolism Markers, RANKL, OPG, and Hormones’ Circulating Levels

Serum levels of osteoprotegerin (OPG), receptor activator of nuclear factor κ-β ligand (RANKL), osteocalcin, osteopontin, parathyroid hormone (PTH) and adenocorticotropin (ACTH) were measured simultaneously using a high sensitivity human cytokine multiplex immunoassay (MilliplexTM MAP, Merck Millipore, Billerica, MA, USA). Assays were run on a Luminex^®^ X-MAP Bio-Plex 200 System Bioanalyzer (Luminex Corp., Austin, TX, USA) according to the kit manufacturer’s instructions. RANKL/OPG ratio was calculated for each subject using the values of RANKL and OPG levels obtained by this method.

### 4.5. Plasma Fatty Acid Profile

Plasma fatty acid profile in rats was determined following Lepage and Roy´s method according to previously described modifications [51].

### 4.6. Plasma Coenzyme Q Determination

Plasma CoQ_10_ levels were assessed by high-performance liquid chromatography (HPLC) combined with electrochemical detection as previously described [52].

### 4.7. Statistical Analysis

Results are expressed as mean ± standard error of the mean (SEM) for six animals. Normal distribution and variance homogenity were evaluated by Kolmogorov–Smirnov and Levene tests, respectively. Variables showing normal distribution were analyzed for differences between dietary treatments at 6 months and at 24 months by analysis of variance (ANOVA) with a Bonferroni post hoc test. Non-normal variables were analyzed by Kruskal–Wallis and Mann–Whitney U non-parametric tests. Tamhane’s T2 test was applied to variables with non-homogeneous variances. To detect significant differences between age groups, for each dietary treatment, the Student’s *t*-test was applied. In all analyses, significant differences were established at *p* < 0.05. Statistical analysis was performed with SPSS 24.0 for Windows (IBM, Chicago, IL, USA).

## Abbreviations

ACTH	Adrenocorticotropin
BMC	Bone mineral content
BMD	Bone mineral density
CoQ_10_	Coenzyme Q_10_
DXA	Dual energy X-ray absorptiometry
EDTA	Ethylenediaminetetraacetic acid
HED	Human equivalent dose
HPLC	High-performance liquid chromatography
MUFA	Monounsaturated fatty acids
n-3 PUFA	n-3 polyunsaturated fatty acids
n-6 PUFA	n-6 polyunsaturated fatty acids
NF-κB	Nuclear factor κB
OPG	Osteoprotegerin
PTH	Parathyroid hormone
PUFA	Polyunsaturated fatty acids
RANKL	Receptor activator of nuclear factor κB ligand
S	Sunflower oil group
SQ	Sunflower oil + CoQ_10_ group
V	Virgin olive oil group
VQ	Virgin olive oil + CoQ_10_ group
WHO	World Health Organization
